# Clinical application and evaluation of metagenomic next-generation sequencing in suspected adult central nervous system infection

**DOI:** 10.1186/s12967-020-02360-6

**Published:** 2020-05-13

**Authors:** Yi Zhang, Peng Cui, Hao-Cheng Zhang, Hong-Long Wu, Ming-Zhi Ye, Yi-Min Zhu, Jing-Wen Ai, Wen-Hong Zhang

**Affiliations:** 1 Department of Infectious Diseases, National Clinical Research Center for Aging and Medicine, Huashan Hospital, State Key Laboratory of Genetic Engineering, School of Life Science, Key Laboratory of Medical Molecular Virology (MOE/MOH) and Institutes of Biomedical Sciences, Shanghai Medical College, Fudan University, 12 Wulumuqi Zhong Road, Shanghai, 200040 China; 2 BGI Genomics, BGI-Shenzhen, Shenzhen, 518083 China

**Keywords:** Metagenomic next-generation sequencing, Central nervous system infection, Application, Etiology, Diagnose

## Abstract

**Background:**

Accurate etiology diagnosis is crucial for central nervous system infections (CNS infections). The diagnostic value of metagenomic next-generation sequencing (mNGS), an emerging powerful platform, remains to be studied in CNS infections.

**Methods:**

We conducted a single-center prospective cohort study to compare mNGS with conventional methods including culture, smear and etc. 248 suspected CNS infectious patients were enrolled and clinical data were recorded.

**Results:**

mNGS reported a 90.00% (9/10) sensitivity in culture-positive patients without empirical treatment and 66.67% (6/9) in empirically-treated patients. Detected an extra of 48 bacteria and fungi in culture-negative patients, mNGS provided a higher detection rate compared to culture in patients with (34.45% vs. 7.56%, McNemar test, p < 0.0083) or without empirical therapy (50.00% vs. 25.00%, McNemar test, p > 0.0083). Compared to conventional methods, positive percent agreement and negative percent agreement was 75.00% and 69.11% separately. mNGS detection rate was significantly higher in patients with cerebrospinal fluid (CSF) WBC > 300 * 10^6^/L, CSF protein > 500 mg/L or glucose ratio ≤ 0.3. mNGS sequencing read is correlated with CSF WBC, glucose ratio levels and clinical disease progression.

**Conclusion:**

mNGS showed a satisfying diagnostic performance in CNS infections and had an overall superior detection rate to culture. mNGS may held diagnostic advantages especially in empirically treated patients. CSF laboratory results were statistically relevant to mNGS detection rate, and mNGS could dynamically monitor disease progression.

## Background

Central nervous system infections (CNS infections) refers to inflammations of brain and spinal cord caused by various pathogenic microbes, including meningitis, encephalitis, abscess and etc. Despite continuously improving diagnostic and treatment skills, CNS infections still compose a considerable portion of human morbidity and mortality, and it is estimated that nearly 320,000 people died from meningitis in 2016 [1]. Timely identification of causative agents is critical for the administration of effective treatment and difficulties in this area still exist. Traditional cerebrospinal fluid (CSF) culture can identify approximately 30–40% CNS infections (including meningitis and encephalitis) [2], and some study even reported 80–90% detection rate in acute community-acquired bacterial meningitis patients [3]. However, in developing countries like China, the detection rate of CSF culture in meningitis ranges 5.4–24.3% from updated surveys [4–7]. The early use of antibiotics in community or emergency department decrease the positive rate of CSF culture. What’s more, lower positive rate in the post-operation meningitis patients might owe to postoperative reaction [6]. Further, CSF culture in blood bottle might improve positive rate, which is not in place in many hospitals. Other diagnostic methods including tissue biopsy, Xpert MTB/RIF Ultra, loop-mediated isothermal amplification (LAMP), and Filmarray meningitis/encephalitis Panel have shown that they could, to some extent, improve diagnostic ability [2, 8], however, these methods are either invasive or are restricted to a limited number of suspected microorganisms.

Recent years have witnessed rapid development of metagenomic next-generation sequencing (mNGS), making it available in the area of precise medicine. mNGS features with simple sample-processing, shorter time, wide range of detectable pathogens and semi-quantitative value in follow-up. To date, several studies have addressed value of mNGS in finding out pathogens and minimizing time of empirical treatment without pathogenic evidence [9–20]. In 2017, mNGS was recommended in ‘Guidelines on the management of infectious encephalitis in adults’ of France as Level 1 evidence to assist clinical decision in CNS infections [21]. However, most previous studies have only focused on clinical cases caused by novel or rare pathogens. Therefore, we conducted an adult prospective study in mainland China to compare diagnostic value between mNGS and conventional methods during CNS infections, and we aimed to elucidate clinical optimization of mNGS in CNS infections as well.

## Materials and methods

### Patients and samples

**Fig. 1:**
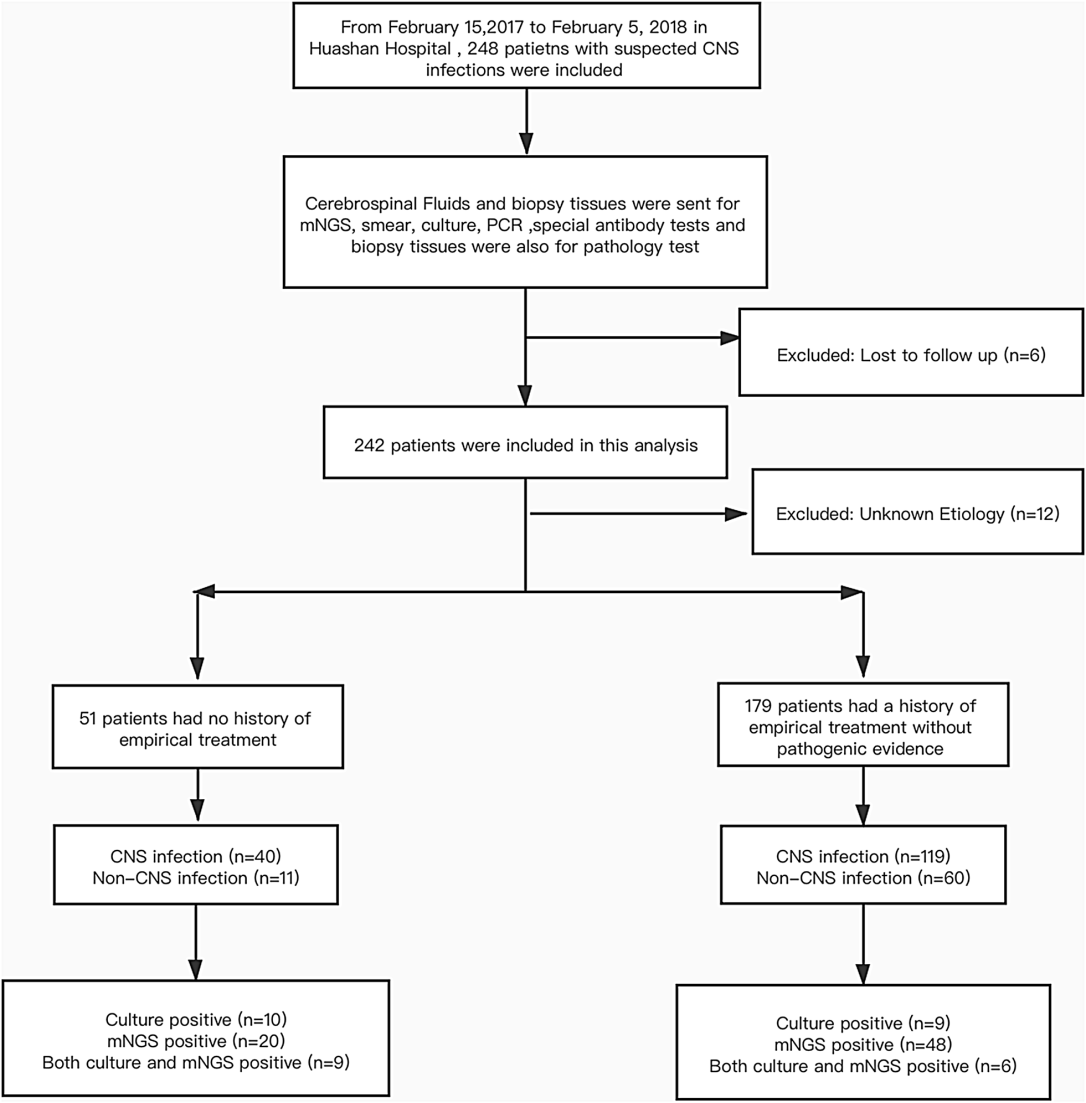
Flowchart for enrollment

From February 15, 2017 to February 5, 2018, we carried out this single-center prospective study with no control group and enrolled suspected CNS infectious patients without pathogenic evidence who were admitted into Huashan Hospital (Fig. 1). Patients or their surrogates signed informed consents and lumbar puncture were done within 24 h of admission. Seven CSF samples were sent for routine and biochemical tests, conventional media culture of bacteria, fungi and tuberculosis, autoimmune antibody and latex agglutination test, and conventional methods including CSF smear, serologic tests, tissue biopsy and nucleic acid amplification testing (NAAT) (traditional PCR, Xpert MTB/RIF, Xpert MTB/RIF Ultra and Filmarray meningitis/encephalitis Panel) were conducted according to clinically assessed necessity. Synchronous CSF sample was collected for mNGS sequencing and patients’ clinical and laboratory data were recorded. Enrollment criteria and composite criteria of final diagnosis of CNS infection were listed in Additional file 1. The primary endpoint of this study was assessment of mNGS diagnostic performance synchronously and the secondary endpoints was clinical optimization of mNGS in CNS infections. Ethical approval was achieved from Huashan Hospital ethical committee.

### Sample sequencing and data analysis

CSF sample was collected by standard procedures. 0.5 mL CSF samples were added with 0.5 mL BioSpec beads (0.5 mm dia. ZIRCONIA/SILICA Cat. No. 11079105z) in the agitation step and DNA was extracted using TIANAmp Micro DNA Kit. DNA libraries were constructed through DNA-fragmentation, end-repair, add A-tailing, adapter-ligation and PCR amplification. A total of 91 patients were suspected as RNA virus CNS infection and reverse transcription and second chain synthesis was performed on their samples during library preparation. RNA extraction was performed on a separate 0.5 mL aliquot from the same CSF sample using QIAamp Viral RNA Mini Kit. Agilent 2100 performed quality control and qualified libraries were sequenced using BGISEQ-100 platform. To control the contamination of each sequencing run, we added a negative control in each run. We duplicated one sample in each run to monitor if the whole process performed well. If the replicates ended in same result, we thought the run performed as expected.

As described before [22, 23], low-quality and short (length < 35 bp) reads were removed. Then the filtered sequences were mapped to human reference database including hg38 and Yanhuang genome sequence using Burrows–Wheeler Alignment (Version: 0.7.10). Remaining data was classified into four Microbial Genome Databases in-house, consisting of viruses, bacteria, fungi, and parasites. Classification reference databases were downloaded from NCBI (ftp://ncbi.nlm.nih.gov/genomes/). RefSeq contains 2700 whole genome sequence of viral taxa, 1494 bacterial genomes or scaffolds, 73 fungi related to human infection, and 47 parasites associated with human diseases. The depth and coverage of each species were calculated with the SoapCoverage soft-ware from the SOAP website (http://soap.genomics.org.cn/). For later analysis, we calculated and ranked absolute and relative abundance for each detected microbe.

$${\text{Absolute Abundance}} = \frac{\text{Reads Number}}{\text{Whole Genome Size}} \times 10^{6}$$

$${\text{Relative Abundance}} = \frac{\text{Absolute Abundance}}{{\mathop \sum \nolimits_{\text{This Sample}} {\text{Absolute Abundance}}}} \times 100{\%}$$

Analysis of the mNGS results included following stages. At least three reads were mapped to the pathogens whose relative abundances should surpass their individual threshold set up by the preliminary sequencing data (Additional file 2: Data Set 1). The preliminary data contains relative abundance of microbes detected by mNGS in health control samples and we set up each microbe’s individual threshold for further test validation: pathogens owned the highest absolute abundance in their genus; pathogens ranked top 10 for bacteria, virus and parasite and ranked top 20 for fungi and *Mycobacterium tuberculosis* in relative abundance after previous two steps of screening (Additional file 3: Dataset 2). After the prior analysis, if the detected pathogens are commonly reported CNS infectious pathogens [24] (Additional file 1: Table S1), they would be considered as causative agents while for non-commonly reported pathogens, mNGS results should be in accordance with the patient’s clinical features or the detected reads would be classified as non-pathogenic microbes sequences. We have uploaded non-human sequences to China National GeneBank (Project accession: CNP0000610).

### Diagnostic assessment of mNGS

We assessed diagnostic performance of mNGS through the following steps. Firstly, we classified participants into two groups in final diagnosis: CNS infections and non-CNS infections using composite criteria (Additional file 1: Table S2). We defined CNS infections as patients who fit clinical criteria with or without etiology criteria (including positive CSF culture of pathogenic microbes and positive result of polymerase chain reaction (PCR) guided by Filmarray meningitis/encephalitis Panel, specific antibody tests and etc.). Second, we calculated mNGS sensitivity compared to culture, mNGS sensitivity compared to culture = mNGS-positive/culture-positive. Then the specificity compared to clinical diagnosis was statistically evaluated, namely specificity compared to clinical diagnosis = mNGS-negative/Clinical diagnosed non-infections. Third, the comparative performance measures of mNGS relative to conventional testing are reported as positive percent agreement and negative percent agreement with the composite criteria. Then, we categorized CNS infections according to different types of causative pathogens (bacteria, virus, fungi and parasite) and further calculated detection rate of mNGS. Moreover, mNGS positive/Case consistent was defined as potential pathogen detected by mNGS that is in consistency with final diagnosis, while mNGS positive/Case inconsistent means inconsistency results between mNGS results and final diagnosis. mNGS negative/Case consistent evidence was provided by consistency between negative mNGS results and final diagnosis at the same time. mNGS negative/Case inconsistent means missed potential pathogen detection of mNGS when comparing to final diagnosis.

### Statistics analysis

For baseline characteristics and CSF laboratory tests, continuous variants were described by means when they conform to the Kolmogorov–Smirnov test and by medians when not. Chi-square test and Fisher’s exact test were used to evaluate independent binomial variables and a p value < 0.05 was considered significantly. In the process of assessing diagnostic performance, p values were adjusted for multiple comparisons using Bonferroni (Dunn) method [8, 25]. A 2-sided McNemar test with significance level of 0.0083 was used to compare differences in diagnostic value of mNGS, culture, conventional methods and combined methods. Further, we did analysis of variance test and Mann–Whitney test to compare differences across mNGS subgroups. Statistical analyses and figures were conducted using the SPSS statistical package 25.0 software and GraphPad Prism 7 software.

## Results

### General characteristics

In total, 248 patients were enrolled, 6 patients were lost to follow-up and 12 patients were not included in the following analysis since their final etiologies were unclear. Among 230 participants, 159 patients were diagnosed with CNS infections, including 125 cases of meningitis and 34 encephalitis patients, and 71 patients were finally diagnosed with non-CNS infectious diseases. In CNS infections, 40 patients didn’t receive empirical treatment before CSF sampling. mNGS and culture were performed in all enrolled samples. Conventional methods excluding culture were carried out in 33 patients according to clinical necessity and 19 patients received positive results. Final CNS infections were categorized into bacterial, fungal, parasitic, viral, mixed and unclassified infections. Non-CNS infections included autoimmune encephalitis, malignant tumor and etc. Baseline characteristics of enrolled patients showed no significant difference among groups (Table 1).

**Table 1:** Baselines characteristics of participants

	CNS infection (159)	Non-CNS infection (71)	p value
CNS infection (n)			
Bacterial infection	95	–	–
Viral infection	38	–	–
Parasitic infection	3	–	–
Fungal infection	10	–	–
Mixed infection^a^	4	–	–
Unclassified	9	–	–
Non-CNS infection(n)			
Autoimmune encephalitis	–	13	–
Malignant tumor	–	13	–
Pulmonary diseases	–	6	–
Tsutsugamushi disease	–	1	–
Local infection	–	7	–
Blood-stream infection	–	1	–
Hematological disease	–	3	–
Rheumatic disease	–	2	–
Psychological disease	–	1	–
Fever of unknown origin	–	7	–
Other neurological disease^b^	–	17	–
Gender(n)			0.791
Male	97	42	
Female	62	29	
Age, year (range)	44.39 (13.00–73.00)	42.30 (15.00–84.00)	0.175
Body temperature, °C (Range)	37.61 (36.20–40.70)	37.47 (36.10–39.60)	0.695
Empirical treatment history(n)			0.103
Yes	119	60	
No	40	11	
Blood laboratory examination (range)			
C-reaction protein, mg/L	19.6 (3.0–126.0)	32.8 (3.0–194.0)	0.568
Erythrocyte sedimentation rate, mm/h	25.20 (2.00–137.00)	26.07 (2.00–118.00)	0.830
Procalcitonin, ng/mL	0.34 (0.01–4.90)	0.19 (0.02–1.82)	0.386
Serum ferritin, ng/mL	631.84 (14.46–2000.00)	560.26 (64.92–1209.00)	0.802
WBC, *10^9^/L	8.84 (2.35–26.03)	9.06 (2.87–27.60)	0.347
Neutrophil, %	69.26 (26.00–95.70)	79.11 (60.4–97.30)	0.427

Statistics: Chi-square for calculations of gender and empirical treatment history, Mann–Whitney test for comparisons in age, body temperature and blood laboratory examination

^a^Mixed pathogens: three patients were defined as bacterial and viral CNS co-infection, and another was defined as bacterial and fungal CNS co-infection

^b^ Other neurological disease: metabolic encephalopathy, hypertrophic cranial pachymeningitis, toxic encephalopathy,intracranial inflammatory granuloma, cerebral venous sinus thrombosis, spinal cord tumor, acute immune encephalomyelitis, acute disseminated encephalomyelitis, cerebral venous sinus thrombosis, demyelinating disease and peripheral neuropathy

### Overall diagnostic performance of mNGS

**Fig. 2:**
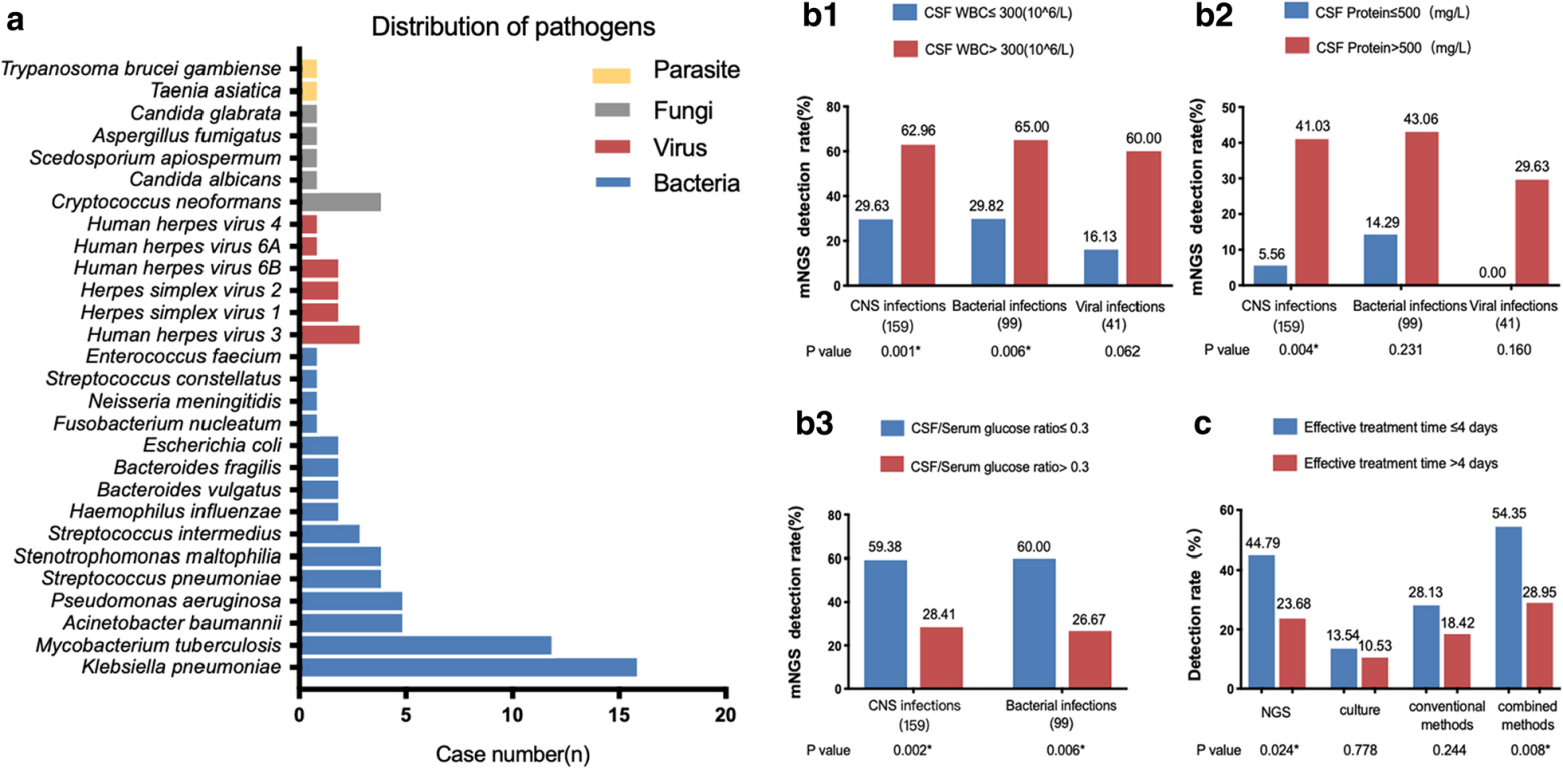
**a** Distribution of pathogens identified by mNGS. **b** The influence of CSF WBC, CSF protein and CSF/Serum glucose ratio on the detection rate of mNGS in CNS infections, bacterial and viral CNS infections defined by composite diagnostic criteria. **c** The influence of effective treatment time on the detection rate of mNGS in CNS infections. Statistics methods (**b**, **c**): Chi-square test

Overall, etiology diagnosis showed that culture reported 19 positive results and mNGS detection revealed that bacteria (n = 61) was the most common identified potential pathogens (Fig. 2a). The top three causative pathogens identified were *Klebsiella pneumoniae*, *Mycobacterium tuberculosis, and Acinetobacter baumannii.* The most detected fungal infection is cryptococcal meningitis. 97 non-pathogenic microbes were detected.

As shown in Table 2, mNGS reported a 90.00% (9/10) sensitivity compared to culture in patients without empirical treatment history and 66.67% (6/9) in patients who had received empirical treatment before admission. The specificity compared to clinical diagnosis of mNGS was 98.59% (70/71). For traditional culture and conventional methods, their specificity compared to clinical diagnosis both reached a 100.00% (71/71).

**Table 2:** The sensitivity of mNGS compared to culture and specificity compared to clinical diagnosis

	Sensitivity compared to culture		Specificity compared to clinical diagnosis
	Without empirical treatment	With empirical treatment	
mNGS	90.00% (9/10)	66.67% (6/9)	98.59% (70/71)
Culture	–	–	100.00% (71/71)
Conventional methods	–	–	100.00% (71/71)

Overall, mNGS owned a 34.45% and 50.00% detection rate of potential pathogens in CNS-infected patients with or without empirical therapy prior to CSF sampling. In the empirically treated patients, the detection rate of mNGS was significantly higher than that of culture and conventional methods (34.45% vs. 7.56%, p = 0.000, McNemar test; 34.45% vs. 18.49%, p = 0.0013 respectively, McNemar test), and combination of mNGS and conventional methods would further improve the causative agent identification. In the non-empirically treated groups, the detection rate of mNGS, culture and conventional methods compared to final diagnosis were not statistically different among each other. Empirical treatment would significantly decrease detection rate of culture, conventional methods (25.00% vs. 7.65%, 40.00% vs. 18.49%, p < 0.05) while that of mNGS was not affected (Table 3).

**Table 3:** The potential pathogen detection rate of mNGS, culture, conventional methods and combined methods in different types of CNS infections

Detection rate	No history of empirical treatment (%, 95% CI)	History of empirical treatment (%, 95% CI)	P value between patients treated or not (%, 95% CI)
Detection rate of CNS infections (n = 159)			
mNGS	50.00% (20/40) (34.06–65.94)	34.45% (41/119)^a^ (26.14–43.79)	0.0802
Culture	25.00% (10/40) (13.25–41.52)	7.56% (9/119)^a^ (3.74–14.27)	0.003
Conventional methods	40.00% (16/40) (25.28–56.61)	18.49% (22/119)^a^ (12.19–26.87)	0.006
Combined methods	55.00% (22/40) (38.66–70.40)	39.50% (47/119) (30.78–48.90)	0.087
Bacterial infections (n = 99)			
mNGS	57.14% (16/28)^b^ (37.43-74.97)	38.03% (27/71)^c,d^ (27.00–50.36)	0.084
Culture	21.43% (6/28)^b^ (9.03–41.46)	11.27% (8/71)^c^ (5.34–21.53)	0.324
Conventional methods	39.29% (11/28) (22.13–59.27)	15.49% (11/71)^d^ (8.35–26.46)	0.010
Combined methods	60.71% (17/28)^b^ (40.73–77.87)	42.25% (30/71)^c,d^ (30.81–54.54)	0.0976
Viral infections (n = 41)			
mNGS	14.29% (1/7) (0.75–57.99)	23.53% (8/34) (11.38–41.57)	> 0.9999
Culture	N/A	N/A	N/A
Conventional methods	0.00% (0/7) (0.00–43.91)	17.65% (6/34) (7.39–35.17)	0.567
Combined methods	14.29% (1/7) (0.75–57.99)	23.53% (8/34) (11.38–41.57)	0.971
Fungal infections (n = 11)			
mNGS	83.33% (5/6) (36.48–99.12)	60.00% (3/5) (17.04–92.74)	0.545
Culture	83.33% (5/6) (36.48–99.12)	20.00% (1/5) (1.05–70.12)	0.080
Conventional methods	100.00% (6/6) (51.68–100.00)	60.00% (3/5) (17.04–92.74)	0.182
Combined methods	100.00% (6/6) (51.58–100.00) (46.29-100.00)	100.00% (5/5) (46.29–100.00)	> 0.9999
Parasitic infections (n = 3)			
mNGS	N/A	66.7% (2/3)	N/A
Culture	N/A	N/A	N/A
Conventional methods	N/A	66.7% (2/3)	N/A
Combined methods	N/A	66.7% (2/3)	N/A

Conventional methods-negative: Conventional methods (culture, smear, special antibody, biopsy and PCR) were reported negative or not conducted

Combined methods: mNGS and conventional methods

Statistics methods: Chi-square or Fisher’s exact test as listed

*N/A* not available

^a^In all CNS infections without history of empirical treatment before admission, the detection rate of mNGS was significantly higher than that of culture and conventional methods (p value < 0.0083, adjusted for multiple comparisons using Bonferroni (Dunn) method)

^b^In bacterial CNS infection patients without history of empirical treatment before admission, the detection rate of mNGS and combined methods were significantly higher than culture (p value < 0.0083, adjusted for multiple comparisons using Bonferroni (Dunn) method)

^c^In bacterial CNS infection patients with history of empirical treatment before admission, the detection rate of mNGS and combined methods were significantly higher than culture (p value < 0.0083, adjusted for multiple comparisons using Bonferroni (Dunn) method)

^d^In bacterial CNS infection patients with history of empirical treatment before admission, the detection rate of mNGS and combined methods were significantly higher than conventional methods. (p value < 0.0083, adjusted for multiple comparisons using Bonferroni (Dunn) method)

**Table 4:** The comparisons of CSF laboratory examinations across mNGS subgroups

	mNGS positive/Case consistent (a)	mNGS positive/Case inconsistent (b)	mNGS negative/Case inconsistent (c)	mNGS negative/Case consistent (d)	p value among all groups (Mann–Whitney test)	p value between group a and c (Mann–Whitney test)	p value between group a and d (Mann–Whitney test)
Suspected CNS infections (a = 61; b = 7; c = 98; d = 70)							
CSF WBC,*10^6^/L (Median(Range))	130 (1–26,000)	60 (1–110)	68 (1–1630)	7 (0–340)	0.001*	0.002*	< 0.0001*
CSF protein, mg/L (Median(Range))	1741.0 (209.0–15,000.0)	839.5 (208.0–5477.0)	1351.0 (33.0–15,000.0)	686.0 (208.0–13,223.0)	0.065	0.002*	< 0.0001*
CSF/serum glucose ratio (Mean (Range))	0.36 (0.06–0.71)	0.37 (0.24–0.5)	0.42 (0.10–0.75)	0.60 (0.13–1.19)	0.023*	0.003*	< 0.0001*
CSF Cl, mmol/L (Mean(Range))	115.65 (97.00–143.00)	119.50 (104.00–130.00)	116.81 (92.00–130.00)	118.52 (96.00–132.00)	0.845	0.110	0.29
CSF pressure, mmH_2_O (Mean(Range))	198.16 (50.00–300.00)	159.40 (70.00–300.00)	194.19 (88.00–300.00)	185.47 (80.00–300.00)	0.598	0.794	0.24
Bacteria (a = 43; b = 5; c = 56; d = 126)							
CSF WBC, *10^6^/L (Median(Range))	138 (1–26,000)	40 (1–110)	68 (4–1630)	20 (0–730)	0.000 *	0.029*	< 0.0001*
CSF protein, mg/L (Median (Range))	1845.5 (209.0–14,224.0)	1112.0 (208.0–2499.0)	1100.0 (33.0–15,000.0)	867.0 (208.0–15,000.0)	0.118	0.115	0.000*
CSF/serum glucose ratio (Mean(Range))	0.29 (0.06–0.52)	0.42 (0.31–0.55)	0.41 (0.19–0.75)	0.53 (0.10–1.19)	0.085	0.001*	< 0.0001*
CSF Cl, mmol/L (Mean(Range))	115.32 (98.00–131.00)	117.20 (104.00–122.00)	114.96 (92.00–126.00)	118.49 (96.00–143.00)	0.680	0.677	0.076
CSF pressure, mmH_2_O (Mean(Range))	212.68 (92.00–300.00)	135.00 (70.00–215.00)	192.48 (88.00–300.00)	188.01 (50.00–300.00)	0.153	0.286	0.114
Virus (a = 9; b = 2; c = 32; d = 187)							
CSF WBC,*10^6^/L (Median(Range))	161 (45–300)	121 (2–242)	35 (1–570)	46 (0–26000)	0.724	0.019*	0.116
CSF protein, mg/L (Median(Range))	1657.0 (662.0–6459.0)	1036.5 (671.0–1456.0)	937.5 (208.0–6741.0)	1066.0 (33.0–15,000.0)	0.190	0.012	0.125
CSF/serum glucose ratio (Mean(Range))	0.44 (0.36–0.59)	0.50 (0.39–0.62)	0.48 (0.31–0.73)	0.45 (0.06–1.19)	0.994	0.299	0.539
CSF Cl, mmol/L (Mean(Range))	117.57 (100.00–131.00)	123.50 (113.00–134.00)	119.61 (104.00–143.00)	116.49 (92.00–132.00)	0.785	0.579	0.527
CSF pressure, mmH_2_O (Mean(Range))	194.17 (100.00–230.00)	140.00 (85.00–195.00)	191.35 (100.00–300.00)	192.28 (50.00–300.00)	0.772	0.935	0.812
Fungi (a = 8; b = 0; c = 3; d = 219)							
CSF WBC, *10^6^/L (Median(Range))	115 (70–730)	N/A	2 (2–15)	44 (0–26,000)	0.902	0.238	< 0.0001*
CSF protein, mg/L (Median (Range))	1122.5 (520.0–15,000.0)		1480.0 (1401.0–1484.0)	1060.0 (33.0–7098.0)	0.145	0.548	0.377
CSF/serum glucose ratio (Mean(Range))	0.33 (0.16–0.52)		0.40 (0.24–0.52)	0.46 (0.06–1.19)	0.774	0.548	0.093
CSF Cl, mmol/L (Mean(Range))	111.83 (97.00–122.00)		122.33 (116.00–130.00)	117.18 (92.00–143.00)	0.684	0.381	0.461
CSF pressure, mmH_2_O (Mean(Range))	170.00 (50.00–300.00)		146.67 (130.00–160.00)	193.15 (70.00–300.00)	0.403	0.786	0.611
Parasite (a = 2; b = 0; c = 1; d = 227)							
CSF WBC, *10^6^/L (Median(Range))	34 (9–58)	N/A	0 (N/A)	50 (0–26,000)	0.947	N/A	0.686
CSF protein, mg/L (Median(Range))	959.0 (506.0–1412.0)		181.0 (N/A)	1079.5 (33.0–15,000.0)	0.682		0.706
CSF/serum glucose ratio (Mean(Range))	0.36 (0.29–0.42)		0.65 (N/A)	0.46 (0.06–1.19)	0.879		0.458
CSF Cl, mmol/L (Mean(Range))	119.50 (111.00–128.00)		127.00 (N/A)	117.04 (92.00–143.00)	0.843		0.703
CSF pressure, mmH_2_O (Mean(Range))	222.50 (150.00–295.00)		135.00 (N/A)	191.13 (50.00–300.00)	0.588		0.697

Statistics analysis: Mann–Whitney test as listed in Table 4

*p value < 0.05

In bacterial CNS infections, detection rate of mNGS was significantly higher than that of culture in both empirically treated and non-empirically treated groups (57.14% vs. 21.43%, p = 0.0016, McNemar test; 38.03% vs. 11.27% p = 0.0001, McNemar test respectively), and empirical antimicrobial treatment would significantly decrease detection rate of conventional methods. No other significant difference was observed in fungal, viral and parasitic infections (Table 3).

### Consistency analysis between mNGS, conventional diagnostic methods and final diagnosis

Overall, mNGS detected an extra of 48 bacteria and fungi in culture-negative CNS infections (Additional file 1: Table S3) and 5 pathogens including 3 *Mycobacterium tuberculosis,* 1 *Candida albicans* and 1 *Escherichia coli* in patients who had negative conventional methods results excluding culture (Additional file 1: Table S4). In 159 CNS infectious patients, mNGS shared a 75.00% and 69.11% identical rate in CSF conventional methods-positive and negative groups (including culture) respectively. In non-CNS infections, mNGS and conventional methods (including culture) demonstrated a 98.59% identical rate and mNGS detected 1 extra pathogen (Additional file 4: Data Set 3).

Among enrolled 230 patients, 61 patients’ mNGS results were classified into mNGS positive/Case consistent group while 70 patients’ mNGS results were categorized into mNGS negative/Case consistent. In culture positive CNS infections, mNGS reported 4 false negative cases including *Cryptococcus neoformans, Bacterium burger,* and *Mycobacterium tuberculosis* (2 cases). mNGS failed to detect 3 *Mycobacterium tuberculosis* cases and 1 *Japanese encephalitis virus* infection in conventional methods positive samples (Additional file 1: Table S4).

7 patients were identified as mNGS positive/Case inconsistent including one report of *Escherichia coli* in non-CNS infection (Additional file 4: Data Set 3), and other 6 mNGS results were considered as both mNGS positive/Case inconsistent and mNGS negative/Case inconsistent, including detection of *Klebsiella pneumoniae* in one tuberculosis meningitis patient, *Haemophilus influenza, Escherichia coli, Acinetobacter baumannii* in three viral meningitis patients respectively and both *Streptococcus constellatus* and *Klebsiella pneumoniae* in one tuberculosis meningitis patient (Additional file 4: Data Set 3).

Further, we compared relative abundances of pathogens (detected more than 5 times) between culture-positive vs. culture-negative and with vs. without empirical treatment patients. Four pathogens including *Klebsiella pneumoniae, Mycobacterium tuberculosis, Acinetobacter baumannii* and *Pseudomonas aeruginosa* were analyzed. The relative abundance of these pathogens in culture-positive patients were higher than that of culture-negative patients, especially in *Klebsiella pneumoniae* (p = 0.0077). For *Mycobacterium tuberculosis* and *Acinetobacter baumannii,* the relative abundance in patients without empirical treatment were higher than that of patients who experienced treatment before admission, while this phenomenon was opposite in the other two pathogens.

### Correlative analysis between mNGS and CSF laboratory results

CSF laboratory results in different CNS infections subgroups revealed that non-CNS infections groups had a significantly lower CSF WBC, protein level and a higher CSF/serum glucose ratio than CNS infection groups, while CSF chlorine and CSF pressure showed no significant differences among different groups (Additional file 1: Table S6).

In suspected CNS infections and its bacterial subgroup, mNGS positive/Case consistent group had a significantly higher CSF WBC and protein levels and a lower CSF/serum glucose ratio than mNGS negative/Case consistent and mNGS negative/Case inconsistent group. Further assessment on influence of CSF laboratory examinations on mNGS detection rate showed that in CNS infections, mNGS detection rate was significantly higher in patients with CSF WBC > 300 * 10^6^/L, CSF protein > 500 mg/L or glucose ratio ≤ 0.3. In bacterial subgroup, CSF WBC > 300 * 10^6^/L and glucose ratio ≤ 0.3 were related with higher mNGS detection rate (Fig. 2b).

### Influence of antimicrobial treatment lengths on diagnostic detection rate of different methods

Although our study had found that empirical treatment would decrease the detection rate of conventional methods (including culture), no significant decrease for the detection rate of mNGS was found in our study. Time lengths of effective antimicrobial treatment analysis was further performed and showed that if patients received effective antimicrobial treatment for more than 4 days before CSF sampling, the detection rate of mNGS would then decrease significantly (44.79% to 22.68%, 54.35% to 28.95%, p value < 0.05) (Fig. 2c).

### Semi-quantitative value of mNGS in the dynamic surveillance of CNS infections

**Fig. 3:**
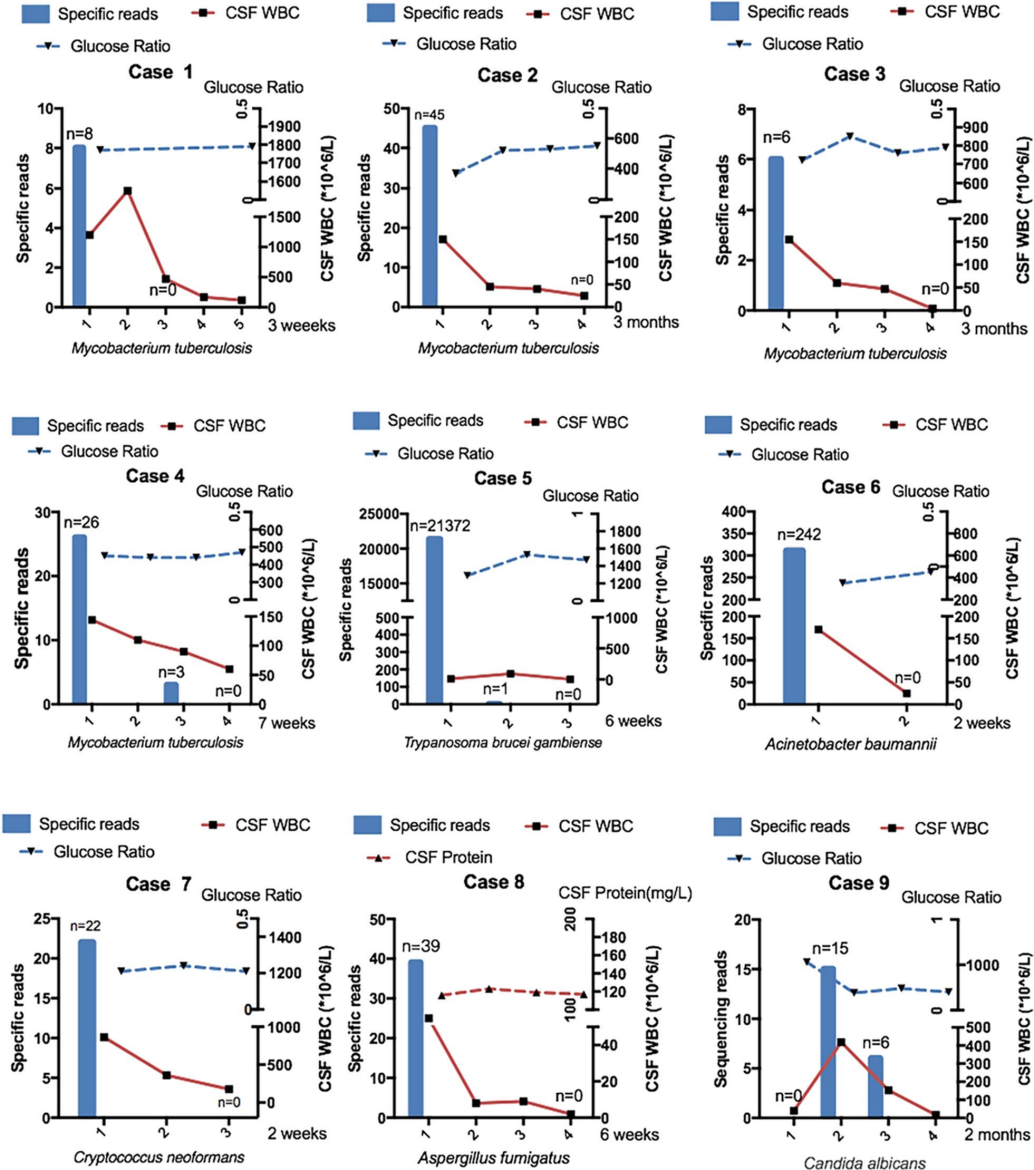
The semi-quantitative value of mNGS in the dynamic surveillance of CNS infections. mNGS sequencing reads was in accordance with synchronously decreased CSF WBC level and increased glucose ratio

We performed repeated mNGS tests on nine cases to observe the dynamic surveillance role of mNGS during CNS infections. Results found direct correlations between mNGS semi-quantitative sequencing reads and CSF WBC and glucose ratio level. In all cases, when patients received effective antimicrobial treatment, mNGS sequencing reads would decline or even turn to negative within weeks, which was in accordance with synchronously decreased CSF WBC level and increased glucose ratio. In case 9, the patient developed *Candida albicans* CNS infection during the hospital stay while treated for autoimmune encephalitis, and therefore, sequencing reads of *Candida albicans* showed a negative–positive–negative conversion during the entire treatment (Fig. 3).

## Discussion

In our study, mNGS detected a wide range of pathogens, including those causative agents commonly and infrequently reported in CNS infections. This demonstrated that our study was capable of reflecting a variety types of diseases that may cause suspected adult CNS infections.

We assessed diagnostic performance of mNGS, culture, conventional methods and combined methods individually compared with clinical final diagnosis. Overall, mNGS detected extra 48 bacteria and fungi in culture-negative patients and 5 potential pathogens in conventional methods-negative tests excluding culture.

For empirically treated patients, mNGS detected significantly more potential pathogens compared to the conventional methods with a detection rate of 34.45%, while for patients without empirical therapy, mNGS also had a 50% detection rate, higher than the 42.50% detection rate using conventional methods. The above results supported previous studies reporting the advantages of mNGS of detecting causative agents in undiagnosed CNS infections cases [26, 27], especially when patients had already received antibiotics treatment.

Also, although mNGS had only a 66.67% and 90% sensitivity compared to culture in the empirically treated and non-treated groups, the ability of which to detect extra pathogens when conventional methods failed suggested that both methods might complement each other to further improve the etiology diagnosis.

This study revealed that for patients who had received empirical antimicrobial treatment before sample collection, mNGS would hold diagnostic advantages over conventional methods. Empirical antibiotics usage would significantly lower detection rate of conventional methods by approximately 20% while that of mNGS was not affected. However, further analysis showed that if delayed-sampling after effective antimicrobial therapy is more than 4 days, it could decrease the mNGS detection rate significantly. The reason behind this phenomenon may likely be due to the different diagnostic mechanisms that lies behind. Previous researches have shown that the detection rate of CSF culture in meningitis could be reduced to 9–11% after effective treatment [28, 29], due to the fact that culture requires the existence of livable pathogens and therefore is easily influenced by the administration of antimicrobial treatment. High-throughput sequencing, on the other hand, need only to identify DNA fragments of microorganisms, which might be the reason for its relatively higher detection rate after treatment. Nevertheless, our study could draw the conclusion that timely sampling of the CSF is a crucial factor in increasing the detection rate during CNS infections.

We further studied diagnostic performance of mNGS in each type of CNS infections. In bacterial CNS infections, mNGS was superior to culture and conventional methods regardless of administration of empirical treatment. For viral infections, mNGS showed similar detection ability compared with serologic tests and NAAT if ordered during clinical approach. Fungal and parasitic CNS infections only had a limited sample size and therefore conclusions could not be drawn from this study.

Compared with CSF conventional methods positive groups, mNGS reported 8 failed cases including 4 *Mycobacterium tuberculosis*, 2 *Cryptococcus neoformans,* 1 *Brucella,* 1 *Japanese encephalitis virus.* One explanation may be that for intracellular bacteria (*Mycobacterium tuberculosis* and *Brucella* etc.), obtaining of their circulatory genome DNA might be more difficult. Fungi had relatively hard-to-break cell walls, which may restrain extraction of pathogen DNA segments. RNA viruses such as *Japanese B encephalitis virus* require reverse transcription before deep sequencing and therefore the amount of the DNA segments might be reduced. In 9 patients clinically diagnosed with tuberculosis meningitis but had negative conventional methods results (PCR or Xpert MTB/RIF), mNGS detected an additional three tuberculosis cases. This suggested that mNGS combined with Xpert MTB/RIF may further raise the detection rate in TBM patients in future.

CSF WBC and CSF/serum glucose ratio showed significant differences between mNGS positive/Case consistent group and mNGS negative/Case consistent group, and this can be easily explained by that elevated CSF WBC and decreased CSF/serum glucose ratio are both associated with higher chance of CNS infections. Another interesting finding revealed that mNGS positive/Case consistent group had a significantly higher WBC level, protein level and lower CSF/serum glucose ratio than mNGS negative/Case inconsistent group. The reason behind may be that a more severe disease condition usually indicates the existence of larger loads of microorganisms, which would then lead to a positive mNGS report. Cut-off value analysis found that patients with CSF WBC > 300 * 10^6^/L, CSF protein > 500 mg/L or glucose ratio ≤ 0.3 will have a significantly higher mNGS detection rates, implying that these patients may be more likely to benefit from mNGS. If taking cost-effectiveness factor into consideration, mNGS may become a prior choice for these patients. Although more host DNA/RNA exist when CSF WBC count increase [20], higher CSF WBC count might indicate a more active diseases status and higher pathogen loads, leading to higher detection rate. To validate our assumptions, we further found direct correlations between mNGS semi-quantitative sequencing reads and CSF WBC and glucose ratio level in Fig. 3, adding proof that mNGS sequencing reads changes were in accordance with synchronously CSF WBC level.

One important character of mNGS is its semi-quantitative value and dynamic change of the value has been proven both in previous study [30] and in our cross-sections to correlate with clinical manifestation of the disease and other laboratory variables. The reason behind may be that a more severe disease condition usually indicates the existence of larger loads of microorganisms, which would then lead to a positive mNGS report. In future, mNGS may provide physicians with a new tool in the direct clinical surveillance of the microorganisms during treatment.

Non-pathogenic microbes’ sequences detected in our study may come from three possible sources: environmental contamination; reagent pollution; errors occurred in sequencing and mapping. We used a negative control in every sequencing run to control contamination and all samples in this study were treated according to the same protocol in the same laboratory.

Our study has some limitations. First, our study had a relatively small sample size of viral, fungal and parasitic CNS infections and therefore could not yet come to conclusions about the diagnostic value of mNGS in these groups. Second, we used preliminary data for analysis but still lack of health control simultaneously. Also, a bactec microbial detection system for CSF culture and novel optimized laboratory and statistical methods for mNGS could be applied to raise positivity [31, 32]. What’s more, RNA library preparations were conducted in a limited number of patients, which might neglect some neuroinvasive RNA viruses. Further, as the mNGS results may be easily influenced by many factors, the standards in our single center cross-section study should be thoroughly modified and tested before applying to other centers.

## Conclusion

This single center study demonstrated that in CNS infected patients, mNGS had an overall superior detection rates of potential pathogens to conventional methods, and the complementation of mNGS and conventional methods would significantly improve the etiology diagnosis. For patients who had received empirical antibiotics treatment, mNGS would hold significant diagnostic advantages over conventional methods, however, the detection rate of mNGS would continuously decrease with the increasing effective treatment lengths. Our findings also discovered that elevated CSF WBC and protein level or decreased glucose ratio is correlated with higher mNGS positivity in CNS infections. Furthermore, mNGS could dynamically surveil pathogen loads and disease progression using semi-quantitative value analysis. Although mNGS has showed the ability to diagnosis clinical infections, it alone still could not replace the necessity of culture. In future, multi-center studies will be needed to explore universal criteria or guidelines of mNGS in CNS infections.

## Supplementary information

**Additional file 1: Table S1.** Commonly reported pathogens in central nervous system (CNS) Infection.** Table S2.** Criteria of CNS infection.** Table S3.** Missed or extra identified bacteria and fungi by mNGS when comparing to culture in CNS infections.** Table S4.** The consistency between mNGS and other conventional methods (PCR, Xpert MTB/RIF, Filmarray, serological antibody test and etc.).** Table S5.** The comparison of relative abundance of pathogens who were detected at least 5 times between a) culture-positive vs culture-negative and b) with vs without empirical treatment patients.** Table S6.** The CSF laboratory examinations among different subgroups.

**Additional file 2: Data set 1.** Preliminary sequencing threshold of relative abundance.

**Additional file 3: Data set 2.** Sequencing details.

**Additional file 4: Data set 3.** Sample details of enrolled participants.

## Data Availability

We have deposited non-human sequences to China National GeneBank Database (CNGBdb) (Project accession: CNP0000610).
