# Supplementary material for: Clinical application and evaluation of metagenomic next-generation sequencing in suspected adult central nervous system infection
Source: J Transl Med. 2020 May 13;18:199. doi: 10.1186/s12967-020-02360-6 (PMC7222471; doi:10.1186/s12967-020-02360-6)
Supplement: Supplementary file 1 — Additional file 1: Table S1. Commonly reported pathogens in central nervous system (CNS) Infection. Table S2. Criteria of CNS infection. Table S3. Missed or extra identified bacteria and fungi by mNGS when comparing to culture in CNS infections. Table S4. The consistency between mNGS and other conventional methods (PCR, Xpert MTB/RIF, Filmarray, serological antibody test and etc.). Table S5. The comparison of relative abundance of pathogens who were detected at least 5 times between a) culture-positive vs culture-negative and b) with vs without empirical treatment patients. Table S6. The CSF laboratory examinations among different subgroups. [file 12967_2020_2360_MOESM1_ESM.docx]

Additional file 1

**Table S1**. Commonly reported pathogens in central nervous system (CNS) Infection

**Table S2.** Criteria of CNS Infection

**Table S3.** Missed or extra identified bacteria and fungi by mNGS when comparing to culture in CNS infections

**Table S4.** The consistency between mNGS and other conventional methods (PCR, Xpert MTB/RIF, Filmarray, serological antibody test and etc.)

**Table S5.** The comparison of relative abundance of pathogens who were detected at least 5 times between a) culture-positive vs culture-negative and b) with vs without empirical treatment patients.

**Table S6.** The CSF laboratory examinations among different subgroups

**Table S1**. Commonly reported potential pathogens in central nervous system (CNS) Infection

| ^Type of pathogens^ | ^Pathogens^ |
| --- | --- |
| ^Bacteria/mycobacteria^ | *^Streptococcus pneumoniae, Neisseria meningitidis, Haemophilus influenzae, Listeria monocytogenes, Group B streptococcus, Escherichia coli, Mycobacterium tuberculosis^* |
| ^Virus^ | *^Enteroviruses, Herpes simplex virus, Varicella zoster virus, West Nile virus, La Crosse virus, Dengue virus, Human herpes virus 6, Lymphocytic choriomeningitis virus, Powassan virus, Rabies virus, Chikungunya virus ,Japanese encephalitis virus, Mumps virus, Human immunodeficiency virus (HIV) ,Adenovirus, Influenza virus^* |
| ^Fungi^ | *^Cryptococcus neoformans , Coccidioides immitis, Blastomyces dermatitidis, Histoplasma capsulatum , Candida spp, Aspergillus spp, Zygomycetes^* ^.^ |
| ^Parasite and other pathogens are rare.^ | |

**Table S2.** Criteria of CNS Infection

| A：Criteria of suspected CNS Infection  Symptoms and signs of central nervous system infection include one or more of the following: fever (>38℃), headache, meningeal signs, vomiting, convulsions, focal neurological deficits, altered consciousness, or lethargy.  And at least one of the following should be met:  a. Increased white cell count, elevated protein, and/or decreased glucose level in CSF.  b. Cerebral imaging indicates pathologic infection changes |
| --- |
| B: Composite criteria of final diagnosis of CNS infection  Central nervous system infection: Clinical criteria with or without etiology criteria.  Non-central nervous system infection: Clinical criteria was not met.  1. Clinical criteria: Symptoms and signs of central nervous system infection include one or more of the following: fever (>38℃), headache, meningeal signs, vomiting, convulsions, focal neurological deficits, altered consciousness, or lethargy.  And at least one of the following should be meet:  a. Increased white cell count, elevated protein, and/or decreased glucose level in CSF  b. Cerebral imaging indicates pathological infection changes  c. Recovered by effective treatment  2. Etiology criteria: At least one of the following methods should be met:  a. There is a positive CSF culture of pathogenic microbes.  b. There is a specific positive result of polymerase chain reaction (PCR) guided by Filmarray meningitis/encephalitis Panel, specific antibody test（diagnostic single antibody titer (IgM) or 4-fold increase in paired sera (IgG)）or pathological examination |

**Table S3.** Missed or extra identified bacteria and fungi by mNGS when comparing to culture in CNS infections

|  | Bacteria and fungi not Identified by mNGS (4) | | Extra bacteria and fungi identified by mNGS (48) | |
| --- | --- | --- | --- | --- |
| Culture-positive CNS Infection  (19) | | *Brucella*  *Cryptococcus neoformans* (2)  *Mycobacterium tuberculosis* | |  |
| Culture-negative CNS Infection  (140) | | N/A | *Mycobacterium tuberculosis(12)；Klebsiella pneumoniae*(12)*; Pseudomonas aeruginosa(3); Streptococcus intermedius(3); Streptococcus pneumoniae(3);* *Haemophilus influenza(2); Bacteroides vulgatus(2); Stenotrophomonas maltophilia(2); Neisseria meningitidis;* *Acinetobacter baumannii; Aspergillus fumigatus; Candida albicans; Cryptococcus neoformans ; Escherichia coli; Fusobacterium nucleatum; Scedosporium apiospermum; Streptococcus constellatus* | |

**Table S4.** The consistency between mNGS and other conventional methods (PCR, Xpert MTB/RIF, Filmarray, serological antibody test and etc.)

|  | | Types of methods | | No. | Pathogens identified by conventional methods (excluding culture) | | Pathogens identified by mNGS | mNGS State | Final diagnosis |
| --- | --- | --- | --- | --- | --- | --- | --- | --- | --- |
| Conventional Methods (excluding culture)  -positive | | Smear (2) | | Case 18 | *Cryptococcus neoformans* | | *Cryptococcus neoformans* | mNGS positive/Case consistent | *Cryptococcus neoformans* CNS Infection |
|  |  |  |  | Case 228 | *Gram-negative bacterium* | | *Haemophilus influenzae* | mNGS positive/Case consistent | *Haemophilus influenzae* CNS Infection |
|  |  | Traditional PCR (3) | Case 36 | | | *Mycobacterium tuberculosis* | *Mycobacterium tuberculosis* | mNGS positive/Case consistent | Tuberculous Meningitis |
|  |  |  | Case 147 | | | *Mycobacterium tuberculosis* | *Mycobacterium tuberculosis* | mNGS positive/Case consistent | Tuberculous Meningitis |
|  |  |  | Case 162 | | | *Human herpes virus 4* | *Human herpes virus 4* | mNGS positive/Case consistent | *Human herpes virus 4* CNS infection |
|  |  | Xpert MTB/RIF(3) | Case 12 | | | *Mycobacterium tuberculosis* | *Klebsiella pneumoniae* | mNGS positive/Case inconsistent &NGS negative/Case inconsistent | Tuberculous Meningitis |
|  |  |  | Case 40 | | | *Mycobacterium tuberculosis* | N/A | mNGS negative/Case inconsistent | Tuberculous Meningitis |
|  |  |  | Case 128 | | | *Mycobacterium tuberculosis* | N/A | mNGS negative/Case inconsistent | Tuberculous Meningitis |
|  |  | Filmarray (6) | Case 54 | | | *Human herpes virus 3* | *Human herpes virus 3* | mNGS positive/Case consistent | *Human herpes virus 3* CNS Infection |
|  |  |  | Case 76 | | | *Human herpes virus 3* | *Human herpes virus 3* | mNGS positive/Case consistent | *Human herpes virus 3* CNS Infection |
|  |  |  | Case 80 | | | *Human herpes virus 6* | *Human herpes virus 6* | mNGS positive/Case consistent | *Human herpes virus 6* CNS Infection |
|  |  |  | Case 140 | | | *Herpes simplex virus 1* | *Herpes simplex virus 1* | mNGS positive/Case consistent | *Herpes simplex virus 1*CNS Infection |
|  |  |  | Case 172 | | | *Klebsiella pneumoniae* | *Klebsiella pneumoniae* | mNGS positive/Case consistent | *Klebsiella pneumoniae* CNS Infection |
|  |  |  | Case 175 | | | *Streptococcus pneumoniae* | *Streptococcus pneumoniae* | mNGS positive/Case consistent | *Streptococcus pneumoniae* CNS Infection |
|  |  | Serological antibody test (2) | Case 49 | | | *Japanese encephalitis virus* (IgM) | N/A | mNGS negative/Case inconsistent | *Japanese encephalitis* |
|  |  |  | Case 118 | | | *Cysticercosis* （CSF IgG） | *Taenia asiatica* | mNGS positive/Case consistent | *Taenia asiatica* CNS Infection |
|  |  | Pathology (1) | Case 110 | | | *Aspergillus fumigatus*  *(brain abscess)* | *Aspergillus fumigatus* | mNGS positive/Case consistent | *Aspergillus fumigatus* CNS Infection |
|  | Other conventional methods (synchronous CSF result negative) (2) | | Case 28 | | | *Penicillium marneffei* （skin pathology） | N/A | mNGS negative/Case inconsistent | *Penicillium marneffei* CNS Infection |
|  |  |  | Case 71 | | | *Trypanosoma brucei gambiense* (bone marrow smear) | *Trypanosoma brucei gambiense* | mNGS positive/Case consistent | *Trypanosoma brucei gambiense* CNS Infection |
| Conventional Methods (exluding culture)-negative | | Traditional PCR (2) | Case 82 | | | N/A | N/A | mNGS negative/Case inconsistent | Tuberculous Meningitis |
|  |  |  | Case 100 | | | N/A | *Candida albicans* | mNGS positive/Case consistent | *Candida albicans CNS Infection* |
|  |  | Xpert MTB/RIF (12) | Case 13 | | | N/A | N/A | mNGS positive/Case inconsistent | Non-CNS Infection |
|  |  |  | Case 29 | | | N/A | *Mycobacterium tuberculosis* | mNGS positive/Case consistent | Tuberculous Meningitis |
|  |  |  | Case 31 | | | N/A | N/A | mNGS negative/Case inconsistent | Tuberculous Meningitis |
|  |  |  | Case 52 | | | N/A | N/A | mNGS negative/Case inconsistent | Tuberculous Meningitis |
|  |  |  | Case 56 | | | N/A | *Mycobacterium tuberculosis* | mNGS positive/Case consistent | Tuberculous Meningitis |
|  |  |  | Case 61 | | | N/A | *Mycobacterium tuberculosis* | mNGS positive/Case consistent | Tuberculous Meningitis |
|  |  |  | Case 138 | | | N/A | N/A | mNGS positive/Case inconsistent | Non-CNS Infection |
|  |  |  | Case 145 | | | N/A | N/A | mNGS negative/Case inconsistent | Tuberculous Meningitis |
|  |  |  | Case 156 | | | N/A | N/A | mNGS negative/Case inconsistent | Tuberculous Meningitis |
|  |  |  | Case 161 | | | N/A | N/A | mNGS positive/Case inconsistent | Non-CNS Infection |
|  |  |  | Case 182 | | | N/A | N/A | mNGS negative/Case inconsistent | Tuberculous Meningitis |
|  |  |  | Case 223 | | | N/A | *Escherichia coli* | mNGS positive/Case inconsistent & NGS negative/Case inconsistent | Virus CNS Infection |

**Table S5.** The comparison of relative abundance of pathogens who were detected at least 5 times between a) culture-positive vs culture-negative and b) with vs without empirical treatment patients.

| **Pathogen** | **Group (a and b question)** | **Relative abundance (median)** | **P value** |
| --- | --- | --- | --- |
| *Klebsiella pneumoniae*  (16) | culture-positive (4) vs culture-negative (12) | 0.52903976  Vs  0.01305041 | **0.0077*** |
|  | History of empirical treatment (6) vs Without history of empirical treatment (10) | 0.13779249  Vs  0.01305041 | 0.0559 |
| *Mycobacterium tuberculosis*  (12) | culture-negative (12) | - | - |
|  | History of empirical treatment (2) vs Without history of empirical treatment (10) | 0.005375667  Vs  0.015195207 | 0.1212 |
| *Acinetobacter baumannii*  (5) | culture-positive (4) vs culture-negative (1) | 7.26E-05  Vs  2.56E-05 | - |
|  | History of empirical treatment (2) vs Without history of empirical treatment (3) | 4.52E-05  Vs  7.10E-05 | 0.8 |
| *Pseudomonas aeruginosa*  (5) | culture-positive (2) vs culture-negative (3) | 0.31103029  Vs  0.04122116 | >0.999 |
|  | History of empirical treatment (2) vs Without history of empirical treatment (3) | 0.32501468  Vs  0.0174749 | 0.4 |

**Table S6.** The CSF laboratory examinations among different subgroups

|  | Bacterial Infection  (99) | Virus Infection  (41) | Fungi Infection  (11) | Parasite Infection  (3) | | Non-CNS Infection  (71) | | P value |
| --- | --- | --- | --- | --- | --- | --- | --- | --- |
| CSF WBC, *10^6^/L(Median(Range)) | 90.00  （1.00-26000.00） | 50.50  （1.00-570.00） | 110.00  （2.00-730.00） | | 33.50  （9.00-58.00） | | 8.00  （1.00-340.00） | <0.001 |
| CSF Protein, mg/L(Median (Range)) | 1418.00  （33.00-15000.00） | 1078.50  (208.00-6741.00） | 1401.00  （520.00-15000.00） | | 959.00  （506.00-1412.00） | | 700.00  （208.00-13223.00） | 0.001 |
| CSF/Serum Glucose Ratio (Mean(Range)) | 0.36  (0.06-0.75) | 0.47  (0.30-0.73) | 0.35  (0.16-0.52) | | 0.36  (0.29-0.42) | | 0.60  (0.13-1.19) | <0.001 |
| CSF Chlorine, mmol/L (Mean (Range)) | 115.11  (92.00-131.00) | 118.63  (100.00-143.00) | 115.33  (97.00-130.00) | | 119.50  (111.00-128.00) | | 118.59  （96.00-132.00） | 0.258 |
| CSF Pressure , mmH₂O (Mean(Range)) | 201.19  (88.00-300.00) | 189.38  (100.00-300.00) | 161.25  (50.00-300.00) | | 222.50  (150.00-295.00) | | 183.25  (70.00-300.00) | 0.627 |
